# Commentary: New evidence for grain specific C_4_ photosynthesis in wheat

**DOI:** 10.3389/fpls.2016.01537

**Published:** 2016-10-19

**Authors:** Parimalan Rangan, Agnelo Furtado, Robert J. Henry

**Affiliations:** ^1^Queensland Alliance for Agriculture and Food Innovation, University of QueenslandBrisbane, QLD, Australia; ^2^Division of Genomic Resources, ICAR-National Bureau of Plant Genetic ResourcesNew Delhi, India

**Keywords:** wheat, seed, C_4_, anatomy, Kranz-anatomy, Bose anatomy

Analysis of the transcriptome revealed the expression of a complete set of enzymes specific for a C_4_ photosynthetic pathway in the pericarp of the developing wheat seed (Rangan et al., 2016). C_4_ photosynthesis is not utilized in wheat leaves with temperatures during the early vegetative stages of wheat growth favoring C_3_ photosynthesis. Wheat crops mature in the spring when growing in the native “Mediterranean” climate of wild wheat progenitors and face rapidly increasing temperatures and drying conditions. The activity of the C_4_ pathway may contribute to crop yields especially in wheat crops under temperature or moisture stress during grain filling. The anatomy of the seed supports photosynthesis with cross cells and tube cells in what we are calling “Bose” anatomy paralleling the “Kranz” anatomy of C_4_ leaves with mesophyll cells and bundle sheath cells. Photosynthesis (C_4_) in these tissues may make a significant contribution to grain yield through efficient carbon capture. C_4_ photosynthesis in the wheat seed provides an adaptation to heat and moisture stress and is an efficient mechanism for minimizing carbon loss due to respiration during grain filling through re-fixation of carbon

New technologies often provide novel perspectives that modify long standing assumptions. The widespread application of modern sequencing (Rossetto and Henry, 2014) to transcriptome analysis has provided unprecedented opportunities for new insights. RNA-Seq of the developing wheat grain has delivered new explanations for the genetic variation in grain quality in wheat (Furtado et al., 2015). Changes in the expression of genes associated with specific metabolic pathways were analyzed at different stages in grain development. The unexpected outcome was the discovery of C_4_ photosynthetic genes in the genome (Rangan et al., 2016) with the expression of C_4_ photosynthetic genes being optimal during early to mid-grain filling. This illustrates the power of genomics in providing novel perspectives in plant biology (Abberton et al., 2016). The developing wheat grain has long been recognized as being photosynthetic but wheat is a classical C_3_ plant. Careful analysis of the literature over a long period of time reveals extensive but highly fragmented evidence for C_4_ photosynthesis in developing wheat grains. Elaborate explanations have often been offered to explain away the results due to the clear evidence for C_3_ photosynthesis in the leaves and the dogma of a requirement for Kranz anatomy for C_4_ photosynthesis to be accomplished. Different pathways may be found in different parts of the same plants or in different environments (Hibberd and Quick, 2002).

Evidence for C_4_ photosynthesis in the pericarp of cereals has been controversial because of uncertainties due to the difficulty of demonstrating flux of carbon through the pathway and the carbon isotope discrimination (Farquhar et al., 1989) being unlike that in C_4_ leaves. The complex pathway of carbon to the pericarp via photosynthetic fixation in C_3_ photosynthesis in the leaves and respiration in the endosperm before arriving at the pericarp explains these issues. Isolated pericarp tissues have been used to demonstrate the flux of carbon into malate and then sugars in barley (Nutbeam and Duffus, 1976).

C_4_ photosynthesis was discovered around 50 years ago and has been associated with adaptation to low carbon environments within the plant often due to high temperatures and the associated management of water loss in dry environments. Plants have been categorized as C_3_ or C_4_ based upon leaf anatomy. Wheat is clearly a C_3_ plant on this basis. However, different photosynthetic pathways have been reported in different organs of plants; and the possibility of C_4_ photosynthesis without Kranz anatomy supports the possibility of grain specific photosynthetic pathways.

The classical C_4_ leaf displays Kranz anatomy allowing the different parts of the C_4_ pathway to be compartmentalized in different cells within the leaf. The inner pericarp of the developing wheat grain has two distinct cell layers, cross cells, and tube cells, that correspond anatomically with the mesophyll and bundle sheath cells of the C_4_ leaf. We propose here that this seed anatomy be described as “Bose” anatomy in honor of the early photosynthesis researcher Jagadish Chandra Bose who reported that C_4_ acids are involved in carbon fixation in *Hydrilla verticillata* during summer (Bose, 1924) and not during winter in 1924 well before the C_3_ pathway of carbon fixation was described in 1957 (Calvin, 1957).

Genes encoding a complete NAD-dependent C_4_ pathway have been detected in the wheat genome and are expressed in the developing wheat grain at the relative levels required to support C_4_ photosynthesis with appropriate subcellular compartmentalization (Rangan et al., 2016). These genes were found in all three progenitor genomes of hexaploid wheat genome suggesting they evolved at a similar time to that reported in other grasses.

Seed photosynthesis is necessarily unique as the seed is a major carbon sink while most photosynthetic tissues are a net source of carbon. This complicates the interpretation of macromolecule assembly may involve significant CO_2_ release. This suggests a role for seed photosynthesis in recapture of CO_2_ generated in the endosperm. Early work by Kriedemann (1966) showed that ear photosynthesis could account for 10–44% of carbon fixation in wheat and that much of the carbon captured by the wheat ear was derived from seed respiration.

Genetic improvement of wheat is critical to the food demands of a growing human population. Targeting selection for enhanced seed photosynthesis may contribute significantly to improving wheat yields. This may be especially important under harsh environmental conditions late in the growth of the crop. Stress late in crop development is often encountered in wheat production and may become increasingly important as a result of climate change (Henry et al., 2016).

## Author contributions

All authors conceived, wrote, and edited the manuscript.

### Conflict of interest statement

The authors declare that the research was conducted in the absence of any commercial or financial relationships that could be construed as a potential conflict of interest.
